# Change in physical activity level and clinical outcomes in older adults with knee pain: a secondary analysis from a randomised controlled trial

**DOI:** 10.1186/s12891-018-1968-z

**Published:** 2018-02-17

**Authors:** Jonathan G. Quicke, Nadine E. Foster, Peter R. Croft, Reuben O. Ogollah, Melanie A. Holden

**Affiliations:** Arthritis Research UK Primary Care Centre, Research Institute for Primary Care and Health Sciences, Keele University, Keele, Staffordshire ST5 5BG UK

**Keywords:** Osteoarthritis, Knee, Pain, Physical activity, Exercise, Geriatrics

## Abstract

**Background:**

Exercise interventions improve clinical outcomes of pain and function in adults with knee pain due to osteoarthritis and higher levels of physical activity are associated with lower severity of pain and higher levels of physical functioning in older adults with knee osteoarthritis in cross-sectional studies. However, to date no studies have investigated if change in physical activity level during exercise interventions can explain clinical outcomes of pain and function. This study aimed to investigate if change in physical activity during exercise interventions is associated with future pain and physical function in older adults with knee pain.

**Methods:**

Secondary longitudinal data analyses of a three armed exercise intervention randomised controlled trial. Participants were adults with knee pain attributed to osteoarthritis, over the age of 45 years old (*n* = 514) from Primary Care Services in the Midlands and Northwest regions of England.

Crude and adjusted associations between absolute change in physical activity from baseline to 3 months (measured by the self-report Physical Activity Scale for the Elderly (PASE)) and i) pain ii) physical function (Western Ontario and McMaster Universities Osteoarthritis Index) and iii) treatment response (OMERACT-OARSI responder criteria) at 3 and 6 months follow-up were investigated using linear and logistic regression.

**Results:**

Change in physical activity level was not associated with future pain, function or treatment response outcomes in crude or adjusted models at 3 or 6 months (*P* > 0.05). A 10 point increase in PASE was not associated with pain β = − 0.01 (− 0.05, 0.02), physical function β = − 0.09 (− 0.19, 0.02) or likelihood (odds ratio) of treatment response 1.02 (0.99, 1.04) at 3 months adjusting for sociodemographics, clinical covariates and the trial intervention arm. Findings were similar for 6 month outcome models.

**Conclusions:**

Change in physical activity did not explain future clinical outcomes of pain and function in this study. Other factors may be responsible for clinical improvements following exercise interventions. However, the PASE may not be sufficiently responsive to measure change in physical activity level. We also recommend further investigation into the responsiveness of commonly used physical activity measures.

**Trial registration:**

(ISRCTN93634563). Registered 29th September 2011.

**Electronic supplementary material:**

The online version of this article (10.1186/s12891-018-1968-z) contains supplementary material, which is available to authorized users.

## Background

Knee pain attributable to osteoarthritis (OA) is both common and disabling in older adults [1]. Exercise and physical activity (PA), including lower limb muscle strengthening and aerobic exercise (for example walking, cycling and swimming) are core recommended treatments in OA clinical guidelines [1–3]. Such interventions are associated with, on average, small to medium effect sizes in terms of reduction in pain and improvements in physical function compared to non-exercise control groups [4–6], although improvements may not be maintained over the longer-term. In order to optimise the effectiveness of exercise interventions, it is important to understand the active components that contribute to improved clinical outcomes [7].

Higher levels of physical activity have been shown to be associated with lower severity of pain and higher physical function in older adults with knee pain in cross-sectional studies [8]. It is plausible that changes in participants’ physical activity level as a result of exercise interventions may explain changes in pain and physical functioning either directly or indirectly. For example, increase in levels of moderate and vigorous cardiovascular intensity physical activity are associated with weight loss in longitudinal cohorts with or at risk of knee OA [9] which is associated with reduced pain and improved function in those who are overweight [10]. However, to date, no studies have investigated the association between change in physical activity level and clinical outcomes of pain and function. The aim of this study, therefore, was to investigate if change in physical activity level is associated with future pain, physical function and overall response to treatment [11] in older adults with knee pain.

## Methods

### Design

This study involved secondary analyses of data from a multi-centre, pragmatic, three-parallel group, randomised controlled trial (RCT) of three physical therapist-led exercise interventions (The Benefits of Effective Exercise for knee Pain-BEEP trial ISRCTN 93634563) [12]. Longitudinal data from all three groups at baseline, three months (following treatment in two of three groups) and six months (following treatment in all groups) were combined, with a priori adjustment for intervention group allocation. Ethical approval was provided by the North West 1 Research Ethics Committee, Cheshire, UK (REC reference 10/H1017/45). Full detail of the RCT is available elsewhere [12] but a concise summary is provided below for context.

### Participants

Participants were adults with knee pain attributable to OA from the BEEP trial (*n* = 514). Inclusion criteria were a clinical diagnosis of knee OA made by either a general practitioner or a primary care research nurse based on age (being 45 years old or older), the presence of pain and/or stiffness in one or both knees [1] and the exclusion of knee pain due to other sources, such as those who had pain due to trauma or recent injury, potentially serious pathology other than OA (such as malignancy or rheumatoid arthritis) and those who had undergone previous total knee replacements. Those with exercise contraindications (such as those with unstable cardiovascular disorders, severe hypertension or congestive heart failure) and those unable to travel to physical therapy treatment centres were also excluded [12].

Participants were recruited from 65 general practices in the midlands and northwest regions of England from: i) records of general practitioner consulters with knee pain in the last year, ii) those referred to physical therapy with knee pain and, iii) adults registered at participating general practices who responded to a questionnaire and reported knee pain [12].

### Trial intervention arms

The three physical therapist-delivered exercise intervention arms were: usual care (UC), individually tailored exercise (ITE) and targeted exercise adherence (TEA). All participants received an advice and information booklet in addition to an exercise programme delivered one-to-one for up to three (UC and ITE) and six months (TEA). Additional intervention detail is provided in Additional file 1: Table S1.

### Outcomes

#### Pain and physical function

Knee pain severity and physical function were assessed using the Western Ontario and McMaster Universities Osteoarthritis Index (WOMAC) pain and function subscales [13]. The pain subscale comprises five items measuring self-reported pain during various activities and gives a total score ranging from 0 (no pain) to 20 (maximum pain). The physical function subscale comprises 17 items and measures the self-reported difficulty an individual has with a broad range of physical functions giving a total score ranging from 0 (no disability) to 68 (maximum disability). Both subscales have been widely used in knee OA studies and their clinimetric properties have been widely investigated elsewhere [13–15].

#### Treatment response

Clinically important response to treatment was assessed using the Outcome Measures in Rheumatology Clinical Trials clinical responder criteria (OMERACT-OARSI responder criteria) [11, 16]. This internationally agreed measure combines outcome data on pain and physical function from the WOMAC scales with patient’s global assessment of change (recorded using a 6 point Likert Scale) [11, 16]. Treatment responders are classified as those who either make a large improvement in pain or function (≥50% improvement and absolute change ≥20 in a 0–100 scale) or a small improvement (≥20% improvement and absolute change≥10 in a 0–100 scale) in two out of three of pain, physical function or global assessment of change (at follow-up compared to baseline).

### Determinants

#### Change in physical activity

Absolute change in physical activity from baseline to three month follow-up (referred to henceforth as *“change in physical activity”*) was calculated by subtracting baseline physical activity score from follow-up physical activity score at three months using the self-report Physical Activity Scale for the Elderly (PASE) [17]. The PASE scale (scored from 0 to over 400 with higher scores indicating higher levels of physical activity) measures physical activity in the previous week summed from questions regarding the frequency and duration of household, leisure time and work physical activity. It has demonstrated construct validity in terms of correlation with 6 min walk test (r = 0.35) and knee strength (0.41) in older adults with knee pain [18] and test-retest reliability in older adults (intra-class correlation 0.75) [17]. The PASE scale has been used in previous longitudinal studies of knee pain and OA [19–21].

### Potential confounders

A range of sociodemographic and clinical covariates measured within the BEEP dataset may be potential confounders of the relationship between change in physical activity and clinical outcomes due to their association with both physical activity [8] and clinical outcome [22, 23]. These included age, gender, Body Mass Index (BMI), individual socioeconomic status [24], employment status, comorbidities (categorised into none, one and two or more), depression measured by the Personal Health Questionnaire (PHQ 8) [25], anxiety measured by the generalized Anxiety Disorder Questionnaire (GAD-7) [26], and widespread pain measured by the Manchester Widespread Pain criteria [27].

### Analyses

All analyses were carried out using STATA version 13.1 [28] and all primary longitudinal analyses utilised a multiply imputed dataset. Multiple imputation (25 imputations) using chained equations was used to adjust for the effect of missing data by maximising sample size and reducing the possible bias associated with loss to follow-up and missing data [29] since there was between 12 and 22% missing clinical outcome data at 3 and 6 months. A wide range of sociodemographic and clinical variables available within the BEEP dataset, including the outcome variables, were used in the imputation model [30].

### Descriptive statistics

Baseline characteristics (complete case) together with longitudinal descriptive statistics (at baseline, three and six months) of physical activity, change in physical activity (measured by the PASE) and clinical outcomes of pain, physical function (WOMAC) and treatment response (OMERACT-OARSI responder criteria) were summarised using means and standard deviations (SD) or frequency and percentage as appropriate.

### Analyses to investigate the association between change in physical activity and clinical outcome

Associations between change in physical activity and clinical outcomes of pain, physical function and treatment response at three and six months were determined using linear and logistic regression. Both univariable and multivariable models were fitted. ANCOVA type multivariable models were used where the clinical outcome variable of interest (pain or function) at 6 months (and subsequently 12 months) were adjusted for baseline clinical severity (pain in the pain and treatment response models, and function in the function model), potential confounders and the trial intervention arm. The a priori decision to hold the intervention arm within multivariable models was made to account for any between trial arm treatment effects (although similar clinical improvements were found in all groups). Additional methodological detail regarding adjusted model building is provided in the Additional file 2.

### Sensitivity analyses

Sensitivity analyses on missing data was performed by use of complete case analysis (CCA), which restricts the analysis to subjects with complete data on the variables involved in the analysis. CCA assumes that missing cases are missing completely at random and makes the assumption that results would be similar to intended sample results [31].

## Results

### Descriptive statistics

Table 1 describes the baseline characteristics of the 514 BEEP trial participants. The sample was 51% female with a participant mean (SD) age of 62.8 (9.7) years. Mean WOMAC pain score (SD) was 8.4 (3.5) and physical function score was 28.1 (12.2) suggesting that on average participants had moderate levels of clinical severity. Mean physical activity score, measured by the PASE, was 177 (83.3) (see Table 2).

**Table 1:** Baseline characteristics

Characteristic	Total (*n* = 514)
Age, *n* (%), years	
45–49	52 (10)
50–59	153 (30)
60–69	183 (36)
70–79	99 (19)
≥80	27 (5)
Female, *n* (%)	262 (51)
BMI, *n* (%), *	
Underweight/ normal	97 (20)
Overweight	208 (42)
Obese	192 (39)
Currently employed n (%) *	214 (42)
Socioeconomic category, *n* (%) *	
Professional	166 (43)
Intermediate	94 (25)
Routine and manual work	124 (32)
Comorbidities, *n* (%)	
None	164 (32)
1 comorbidity	180 (35)
2 or more comorbidities	170 (33)
PHQ 8, 0–24, mean (SD) *	4.0 (+/− 4.7)
WOMAC, mean (SD)	
Pain, 0–20, *	8.4 (+/− 3.5)
Function, 0–68, *	28.1 (+/− 12.3)
Stiffness, 0–8, *	3.7 (+/− 1.7)
Knee pain duration, *n* (%), years *	
≤ 1	125 (25)
More than1 but < 5	198 (39)
More than 5 but < 10	94 (19)
10+	91 (18)
Widespread pain *n* (%) *	79 (15)

*Footnote***:** Baseline descriptive statistics based on complete cases; * = subject to missing data (hence individual item frequencies may not add to total sample). Missing data was 2% in primary clinical variables, less than 10% in all remaining variables except socioeconomic category which was 25% missing. Body Mass Index: less than 25 = underweight/ normal, 25 or more but less than 30 = overweight, 30 or more = obese. Comorbidities included (in descending order of frequency) Hypertension, Asthma, Diabetes, Angina, Heart attack and Heart failure

*Abbreviations*: *BMI* Body Mass Index, *PHQ* 8  Personal Health Depression Questionnaire (higher scores indicate lower mood), *SD*  Standard deviation; Widespread pain = Manchester Widespread Pain [27]; *WOMAC* Western Ontario and McMaster Universities Osteoarthritis Index

**Table 2:** Physical activity and clinical outcome longitudinal summary statistics

Variables (range)	Baseline	3 months	6 months
PASE (0–400+)	177.0 (83.3)	192.1 (87.9)	190.5 (89.3)
WOMAC pain (0–20)	8.4 (3.5)	6.7 (3.6)	6.3 (3.9)
WOMAC function (0–68)	28.1 (12.2)	23.6 (12.5)	21.7 (13.7)
OMERACT-OARSI response (%)	NA	45	52

*Footnote***:** Multiple imputed data (combined results from 25 imputed datasets). All values are mean scores (standard deviation) except OMERACT-OARSI response which are given in percentages. All scores indicate higher levels of the variable except WOMAC function with higher scores indicating lower functioning

*Abbreviations*: *OMERACT-OARSI* Outcome Measures in Rheumatology Clinical Trials-Osteoarthritis Research Society International, *PASE*  Physical Activity Scale for the Elderly, *WOMAC* Western Ontario and McMaster Universities Osteoarthritis Index

The proportion of the baseline sample lost to follow up was 17% at three months and 11% at six months. For information, participants lost to follow-up had slightly worse pain and function at baseline, higher levels of depression and anxiety at baseline and were less likely to have used facilities for physical activity in the last 7 days [30]. Mean PASE increased to 192.1 (87.9) at three month follow-up and then remained relatively stable at six month follow-up 190.5 (89.3). Clinical outcome scores of pain and function improved over time with 45% classified as treatment responders at three months and 52% at six months follow-up. At three months, mean WOMAC pain and function were 6.7 (3.6) and 23.6 (12.5) respectively, changing to 6.3 (3.9) and 21.7 (13.7) at six month follow-up (see Table 2).

The mean PASE change score between baseline and three months was an increase of 15.1 with substantial individual variation as indicated by a standard deviation of 87.4. Change scores for physical activity and clinical outcomes are reported in Table 3.

**Table 3:** Physical activity, pain and function change scores

Change variable	Mean score baseline to 3 months(SD)	Mean score baseline to 6 months(SD)
Change in PASE	15.1 (87.4)	13.5 (86.9)
Change in WOMAC pain	−1.6 (3.2)	−2.1 (3.5)
Change in WOMAC function	−4.5 (10.1)	−6.4 (11.8)

*Footnote***:** Multiple imputed data (combined results from 25 imputed datasets); all change scores calculated by subtracting the score at baseline from the score at three or six month follow up; Higher change in PASE scores indicate higher physical activity at follow up compared to baseline; Negative change in WOMAC pain and function scores indicate reduced pain and higher function at follow-up compared to baseline

*Abbreviations***:** *PASE*  Physical Activity Scale for the Elderly, *WOMAC* Western Ontario and McMaster Universities Osteoarthritis Index

### Associations between change in physical activity and clinical outcome

Univariable models showed change in physical activity was not significantly associated with any of the three clinical outcomes of pain, physical function (Tables 4 and 5) or treatment response at both three and six months (*p* > 0.05) (Table 6).

**Table 4:**
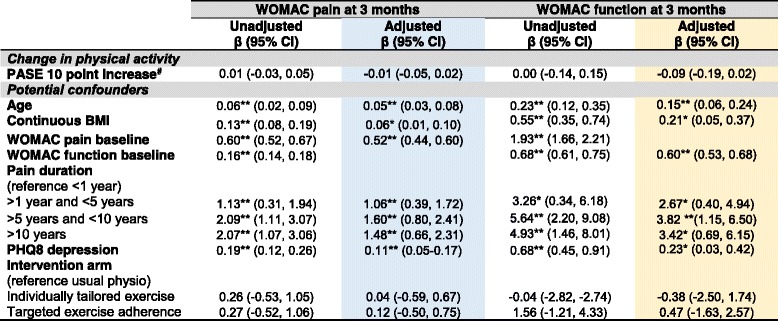
The association between change in physical activity level and pain and function at three months follow-up

Key**:** White = Unadjusted Models; Blue = Adjusted pain at 3 months model; Gold = Adjusted physical function at 3 months model

*Footnotes***:** Multiple imputed data; multiple linear regression adjusted models selected via backwards elimination holding treatment arm and change in physical activity in the model. * = statistically significant β coefficient *P* < 0.05; ** = statistically significant β coefficient *P* < 0.01. Higher WOMAC scores indicate higher pain and worse function. Higher PASE score indicates higher level of physical activity, ^#^absolute change in PASE calculated by subtracting the baseline score from the score at three months. Higher PHQ8 depression scores indicate worse depression

*Abbreviations*: *β*  Unstandardized coefficients, *BMI* Body Mass Index; CI=Confidence Interval, *PASE*  Physical Activity Scale for the Elderly, *PHQ8*  Personal Health Questionnaire, *WOMAC* Western Ontario and McMaster Osteoarthritis Index

**Table 5:**
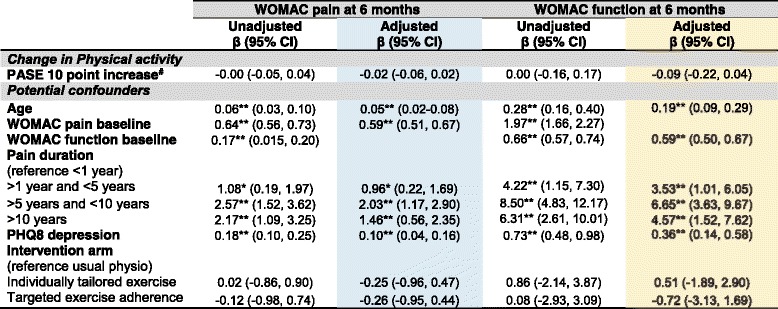
The association between change in physical activity level and pain and function at six months follow-up

Key**:** White = Unadjusted Models; Blue = Adjusted pain at 6 months model; Gold = Adjusted physical function at 6 months model

*Footnotes***:** Multiple imputed data, multiple linear regression adjusted models selected via backwards elimination holding treatment arm and change in physical activity in the model. Regression coefficients shown are rounded to two decimal places and a score of − 0.00 is used to indicate a very small yet negative confidence interval coefficient. * = statistically significant β coefficient *P* < 0.05; ** = statistically significant β coefficient *P* < 0.01. Higher WOMAC scores indicate higher pain and worse function. Higher PASE score indicates higher level of physical activity, ^#^absolute change in PASE calculated by subtracting the baseline score from the score at three months. Higher PHQ8 depression scores indicate worse depression

*Abbreviations*: *β*  Unstandardized coefficients, *BMI* Body Mass Index, *CI* Confidence Interval, *PASE*  Physical Activity Scale for the Elderly, *PHQ8* = Personal Health Questionnaire, *WOMAC* Western Ontario and McMaster Osteoarthritis Index

**Table 6:**
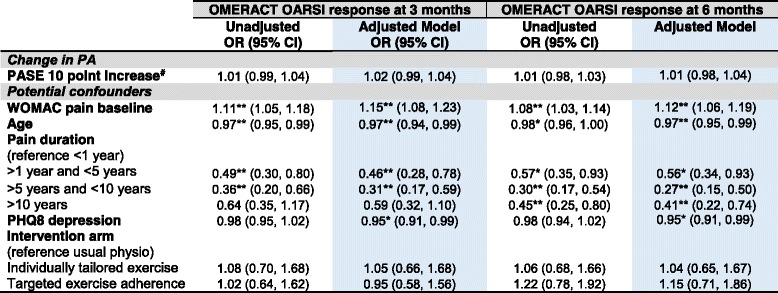
The association between change in physical activity level and treatment response at three and six months follow-up

Key**:** White = Unadjusted Models; Blue = Adjusted OMERACT-OARSI treatment response models

*Footnotes*: Multiple imputed data, multiple logistic regression adjusted models selected via backwards elimination holding treatment arm and change in physical activity in the model. Higher WOMAC scores indicate higher pain. * = statistically significant OR P < 0.05; ** = statistically significant OR P < 0.01. Higher PASE score indicates higher level of physical activity, ^#^absolute change in PASE calculated by subtracting the baseline score from the score at three months. Higher PHQ8 depression scores indicate worse depression

*Abbreviations*: *β*  Unstandardized coefficients, *CI* Confidence Interval, *OMERACT OARSI* Osteoarthritis Research Society International set of responder criteria for osteoarthritis clinical trials, *PASE*  Physical Activity Scale for the Elderly, *PHQ8*  Personal Health Questionnaire, *WOMAC* Western Ontario and McMaster Osteoarthritis Index; yr. = year

Change in physical activity remained non-significantly associated with clinical outcomes in all multivariable clinical outcome models adjusting for age, continuous BMI, pain duration, depression, the trial intervention arm and baseline pain/function (*P* > 0.05) (see Tables 4, 5 and 6). An increase of 10 PASE points between baseline and three months had a non-statistically significant, adjusted association with WOMAC pain at three (β = − 0.01 (95% Confidence Interval − 0.05, 0.02)) and six (β = − 0.02 (− 0.06, 0.02)) months. Interpreting these best estimate β coefficients, for every increase in physical activity of ten points on the PASE, WOMAC pain score decreased by 0.01 at three months and 0.02 at six months. These results are not statistically significant since the 95% confidence intervals cross zero. Similarly, an increase of 10 points on the PASE scale had a non-statistically significant adjusted association with function at three β = − 0.09 (− 0.19, 0.02) and six months β = − 0.09 (− 0.22, 0.04) and, treatment response at three months OR = 1.02 (0.99, 1.04) and six months OR = 1.01 (0.98, 1.04). Interpreting the treatment response best estimate odds ratios, for every increase in physical activity of ten points on the PASE, participants had a 2% increase in the odds of being able to be classified as a treatment responder using the OMERACT-OARSI criteria at three month follow-up and 1% increase in odds at six month follow-up but these findings were not statistically significant since the 95% confidence interval odds ratios cross one.

Complete case sensitivity analyses, investigating the adjusted association between a 10 point increase in PASE and clinical outcomes of WOMAC pain and function at 3 and 6 months produced similar non-statistically significant associations (results not shown).

## Discussion

### Main findings

This study investigated whether change in physical activity was associated with future clinical outcomes of pain and physical function in older adults with knee pain attributable to OA. This question is novel and important since change in physical activity may explain clinical improvements following exercise interventions and inform future interventions. The main finding from this RCT was that change in physical activity level was not associated with future pain, physical functioning or treatment response at either three or six month follow-up. Small β coefficients were expected given the difference in scale between the PASE (0–400+) and WOMAC pain and function scores (0–20 and 0–68 respectively) (since the PASE scale is larger by approximately a factor of 20 than the WOMAC pain scale). However, even taking this in to account and allowing for a 10 point change in PASE, the magnitude of associations were very small, non-statistically significant and do not appear to be of clinical importance (Tables 4, 5 and 6). For example, extrapolating from the β coefficients, changing physical activity by a full standard deviation (83 points on the PASE) would be associated with less than a 1 point change in WOMAC pain or function at either three or six months. Similarly, large changes in physical activity would only have a very small effect on the odds of being an OMERACT-OARSI responder.

The null association findings (Tables 4, 5 and 6) suggest that change in overall general physical activity level, as measured by the PASE, does not explain clinical outcome following exercise intervention within the BEEP trial and that other variables may be responsible for the observed improvements in pain and function (see Table 2). For example, lower limb strengthening [32] or psychosocial factors (such as outcome expectations, attention and monitoring, the interest and empathy expressed by physiotherapists and the credibility of the intervention) may contribute to improvements in pain and function [33, 34].

There is also a separate or further explanation for the null findings. Measuring change in physical activity using the self-report PASE involves a number of limitations that may increase the chance of a Type II error (i.e. rejecting an association between change in physical activity and clinical outcome if one exists). Although the PASE has been highlighted as a promising measure of physical activity in older adults with OA [35], all self-report measures of physical activity can either over- or under- estimate actual physical activity level [36] since they are at risk of recall bias (through errors in memory and activity recall), misclassification of physical activity intensity and duration [15, 37], and social desirability bias by participants [38]. Furthermore, modelling change in physical activity is methodologically challenging and using an absolute change score between two time-points may compound measurement errors and reduce regression coefficient precision, biasing our findings towards the null [39].

The PASE minimal detectable change statistic (87), which measures the threshold for a “real” change that with 95% confidence is beyond measurement error [40], is considerably larger than the mean change detected in the BEEP dataset (15.1). This suggests that the mean change in PASE scores detected during the exercise interventions was relatively small, potentially affected by measurement error and perhaps insufficient to influence clinical outcomes or alternatively that the PASE may have inadequate responsiveness in older adults with joint pain.

To the authors’ knowledge, this is the first study to explicitly investigate the association between change in physical activity level and clinical outcomes of pain and function in older adults with knee pain attributable to OA. However, similar studies have investigated the association between change in physical activity level and disability in those with low back pain [41] and pain severity and physical function outcomes in those with fibromyalgia [42, 43]. Similar to our findings, no association was found in the back pain study using either self-report or accelerometer measured physical activity [41], however, associations were found between change in physical activity and future clinical outcomes in the two fibromyalgia studies [42, 43]. Whilst the aetiological differences between knee pain attributed to OA and fibromyalgia are likely to be substantial and prevent direct comparison, these findings do suggest it is possible to detect associations if they exist between self-report change in physical activity and clinical outcomes despite the previously discussed challenges in measuring change in physical activity.

### Strengths and limitations

The strengths of this study were the ability to investigate both univariable and adjusted associations between change in physical activity and future clinical outcomes at two separate time-points. The use of multiple imputation helped preserve sample size, reducing the risk of bias due to loss to follow-up [29] whilst the sensitivity analysis added strength to the primary findings by exploring the dataset under different missing data assumptions.

The main study limitation, relating to the challenges in measuring change in physical activity, has been discussed above. With our available data we were unable to investigate levels of different types of physical activity, for example, time spent in strengthening or different cardiovascular intensities of exercise. Different types of physical activity may have had different effects on outcome. Another concern for our analysis is temporal bias. Temporal bias occurs when the inference about proper temporal sequence of cause and effect is erroneous [44]. Attempts were made to measure the exposure of interest- change in physical activity level (baseline to three months) prior to the clinical outcomes (at three and six months). Nevertheless, change in pain or physical function may have occurred prior to any change in physical activity, meaning we cannot be sure about the direction of any potential cause and effect. In handling missing data using multiple imputation for our analysis, we made the assumption that our data was “missing at random” (MAR) [29] since we deemed it likely that missing values could be estimated from observed values. If any missing data was “missing not at random” (MNAR) i.e. there are systematic differences between missing and observed values even after the observed data are taken into account then our multiple imputed analysis would be at risk of bias [29].

Finally, before generalising our findings it is important to remember this sample population were older adults with knee pain attributed to clinically diagnosed OA from a RCT of exercise interventions. Other populations of older adults with knee pain, for example, those who did not consent to exercise interventions may have different clinical outcomes with changing their physical activity levels.

### Implications

Although change in physical activity was not shown to be associated with clinical outcomes of pain and physical function, insufficiently active older adults with knee pain can still be advised to increase their physical activity levels as able, in order to achieve the associated general health benefits [45–48] with the reassurance that modest increases in physical activity are not associated with increasing pain or deterioration in function at a group level.

In order to select the most appropriate physical activity measure for longitudinal studies measuring change in physical activity in older adults with knee pain, future research could investigate and compare the reliability and responsiveness of the PASE alongside other recommended measures of physical activity such as the International Physical Activity Questionnaire (IPAQ) [37] and direct measures such as pedometry and accelerometry.

The inclusion criteria for this trial sample was based on older adults with knee pain regardless of baseline level of physical activity. Future studies could investigate specific subgroups of older adults with knee pain, such as those who are inactive, who may plausibly respond differently to increases in physical activity than all adults with knee pain or those currently meeting guideline recommended activity levels.

## Conclusions

Change in physical activity level was not associated with future pain, physical function or the proportion of participants who could be classified as treatment responders in this secondary analysis of a randomised trial dataset. Hence other factors may be responsible for improvements in these clinical outcomes following exercise interventions. Accurately measuring change in physical activity in older adults with knee pain remains a challenge and the PASE, although useful for capturing population snap shots of physical activity level, may not be sufficiently responsive to detect changes over time in this clinical population. We recommend the further investigation of responsiveness in commonly utilised measures of physical activity for older adults with joint pain including the PASE to aid future longitudinal studies assessing change in physical activity.

## Additional files

Additional file 1: **Table S1.** Summary of the BEEP trial interventions (Foster et al. [12]). (DOCX 15 kb)

Additional file 2 Supplementary material: Adjusted model building detail. (DOCX 15 kb)

## Abbreviations

BEEP: Benefits of Effective Exercise for Knee Pain
BMI: Body Mass Index
GAD-7: Generalised Anxiety Disorder-7
IPAQ: International Physical Activity Questionnaire
ISRCTN: International Standard Registered Clinical/soCial sTudy Number
ITE: Individually Tailored Exercise
MDC: Minimal Detectable Change
NIHR: National Institute for Health Research
OA: osteoarthritis
OMERACT-OARSI: Outcome Measures in Rheumatology Clinical Trials-Osteoarthritis Research Society International
OR: odds ratio
PA: Physical activity
PASE: Physical Activity Scale for the Elderly
PHQ 8: Personal Health Questionnaire 8
RCT: Randomised controlled trial
REC: Research Ethics Committee
TEA: Targeted Exercise Adherence
UC: Usual Care
WOMAC: Western Ontario and McMaster Universities Osteoarthritis Index
