# Supplementary material for: Change in physical activity level and clinical outcomes in older adults with knee pain: a secondary analysis from a randomised controlled trial
Source: BMC Musculoskelet Disord. 2018 Feb 17;19:59. doi: 10.1186/s12891-018-1968-z (PMC5816451; doi:10.1186/s12891-018-1968-z)
Supplement: Supplementary file 1 — Table S1. Summary of the BEEP trial interventions (Foster et al. [12]). (DOCX 15 kb) [file 12891_2018_1968_MOESM1_ESM.docx]

**Additional file 1: Table S1. Summary of the BEEP trial interventions (Foster et al 2014)**

| **Key features** | **Usual Physiotherapy Care** | **Individually Tailored Exercise** | **Targeted Exercise Adherence** |
| --- | --- | --- | --- |
| **Number of sessions**  **Time period of treatment**  **General education**  **Exercise focus**  **Individualisation**  **Progression**  **Supervision**  **Exercise monitoring**  **Provision of follow-up** | Up to 4  Up to 12 weeks  Advice and information booklet  Lower limb exercise  Exercises selected from a standardised written template  Minimal progression  Minimal supervision  No exercise diary  None after 12 weeks | 6 to 8  Up to 12 weeks  Advice and information booklet  Lower limb exercise  Exercises individually prescribed for each patient, supported by an individualised, written exercise programme  Good progression  Good supervision  Exercise diary  None after 12 weeks | 8 to 10  Up to 6 months  Advice and information booklet  Lower limb and general exercise. Signposting and support to engage in local community based general physical activity opportunities.  Exercises individually prescribed for each patient, supported by an individualised, written exercise programme    Good progression  Good supervision  Exercise and physical activity diaries  Follow-up and monitoring contacts (telephone or face to face) through to 6 months |
