# Supplementary material for: Change in physical activity level and clinical outcomes in older adults with knee pain: a secondary analysis from a randomised controlled trial
Source: BMC Musculoskelet Disord. 2018 Feb 17;19:59. doi: 10.1186/s12891-018-1968-z (PMC5816451; doi:10.1186/s12891-018-1968-z)
Supplement: Supplementary file 2 — Supplementary material: Adjusted model building detail. (DOCX 15 kb) [file 12891_2018_1968_MOESM2_ESM.docx]

**Supplementary material: Adjusted model building detail**

Model building for multivariable models was carried out in three distinct stages. Stage one explored correlations between pairs of potential confounders followed by the removal of one variable from each pair that were highly correlated (Pearson’s correlations higher than 0.7), based on clinical importance and previous evidence of association with clinical outcome, to reduce the problem of collinearity within adjusted models (Tu et al 2005).

Stage two of model building involved entering change in physical activity and all remaining potential confounders into a multiple linear regression (pain and physical function models) or logistic regression model (OMERACT-OARSI model). Change in physical activity, baseline clinical severity (pain in the pain outcome and OMERACT-OARSI models, function in the function outcome model) were held within the model a priori, followed by iterative manual backwards elimination of potential confounders with non-significant regression coefficients (>0.05) until all remaining variables were significant within the model (Kutner et al 2005).

Stage three involved model assumption checking, further collinearity checking using the Variance Inflation Factor Statistic (within multiple linear regression models) and checking for post-hoc model overfit using a conservative estimate of 10 participants per variable within the model (Kutner et al 2005, Babyak 2004).

**Supplementary references:**

Tu Y-K, Kellett M, Clerehugh V, Gilthorpe MS. Problems of correlations between explanatory variables in multiple regression analyses in the dental literature. *British Dental Journal*. 2005;199(7):457–61.

Kutner MH. *Applied Linear Statistical Models*. 5th edition. London: McGraw-Hill Irwin; 2005.

Babyak MA. What you see may not be what you get: a brief, nontechnical introduction to overfitting in regression-type models. *Psychosomatic Medicine*. 2004;66(3):411–21.
